# Supplementary material for: TELEHEALTH and digital health platforms in promoting access to sexual reproductive health self care among youth: A case of rocket health services in Uganda
Source: PLOS Digit Health. 2025 Oct 31;4(10):e0000770. doi: 10.1371/journal.pdig.0000770 (PMC12578243; doi:10.1371/journal.pdig.0000770)
Supplement: Attachment — Submitted filename: Response to Reviewers.pdf [file pdig.0000770.s002.pdf]

Makerere University School of Public Health
Department Health Policy, Planning and Management

July 14 2025,

Haleh Ayatollahi

Section Editor

PLOS Digital Health,

Resubmission: ***<u>TELEHEALTH and digital health platforms in promoting access to sexual reproductive health self care among youth: A case of Rocket health services in Uganda</u>***

Dear Haleh Ayatollahi,

Thank you for the opportunity to revise our manuscript, "*TELEHEALTH and digital health platforms in promoting access to sexual reproductive health self-care among youth: A case of Rocket health services in Uganda*" PDIG-D-25-00072 for *PLOS* Digital Health.

We have addressed the reviewers' and editors' comments in our responses on the following pages and incorporated related changes into the manuscript. We appreciate the feedback and believe our manuscript is notably improved by our revisions following the reviewers' thoughtful evaluation.

This manuscript explores the different digital health platforms and how they promote access to reproductive health self-care among youth

These findings are pivotal for informing policymakers as they strategize policies, research, and resource allocation to Sexual Reproductive Health and self-care. We believe that publishing this work in PLOS Digital Health will significantly enhance its visibility and impact on policy decisions worldwide

We appreciate your consideration of this important manuscript and are eager for the opportunity to contribute to the body of research published by PLOS Digital Health.

Warm regards,

Vincent Ssenfuka
ssenfukavicent@gmail.com

Reviewer 1

1. The abstract follows a logical flow, clearly presenting the background, methods, results, and conclusions of Telehealth and Digital health platforms.

Response: Thank you

2. The findings offer actionable insights into digital health platforms, which can inform policymakers and healthcare providers on improving SRH access.

Response: Thank you

3. The study could benefit from a comparison with other digital health platforms or similar initiatives in different regions to contextualize its findings.

Response: Comparisons have been added in the discussion section

4. The abstract presents several percentages and numbers, but there is no indication of statistical significance or confidence intervals. While not always necessary in an abstract, mentioning key trends or significant differences could add depth.

Response: Statistical significance was not applied in this study since the primary objective was descriptive and explanatory in nature rather than to test hypothesis or infer causal relationships. The analysis aimed to summarize service utilization patterns among youth accessing SRH service through digital health platforms. The proportions were supported by qualitative insights to offer comprehensive understanding of the user behaviour and system level dynamics. Future analytical studies with defined control groups may be better suited for statistical comparisons.

5. The conclusion mentions "targeted interventions" but does not outline what these interventions might be (e.g., subsidy programs, awareness campaigns, or digital literacy training). A brief mention would strengthen the impact.

Response: Thank you very much for this comment. We have mentioned some of these interventions in the abstract, line 44

6. The article focuses more on digital health platforms and their usage rather than on telehealth services that support patients in need. Incorporating telehealth services would strengthen the article.

Response: Thank you for this valuable comment. We agree that telehealth services are a critical component of digital health in supporting patient care. This was incorporated in the manuscript. Specifically, we highlighted Rocket Health's use of teleconsultations as part of its service delivery model which is essential in providing personalized support, clinical advice and prescription services.

7. The statement that sildenafil is the most consumed product suggests a significant demand for erectile dysfunction (ED) treatments, but it lacks context.

Is this increase due to an actual rise in ED prevalence among young men, greater social acceptance of seeking treatment, or aggressive marketing by digital platforms?

Response: This has been rectified in the discussion section 523-526

8. Additionally, some young men use sildenafil recreationally rather than for diagnosed ED. This distinction is important for interpreting the data accurately.

Response: This has been addressed in the discussion section line 523-526

9. This should be included as a downside of easy access to these medications on online platforms, as it may bypass addressing the root cause and seeking proper medical attention.

Response: Thank you for the suggestion, this has been included, line 528-533

10. The review acknowledges that digital platforms facilitate access to SRH products, but it does not explore whether they also play a role in education, counseling, or misuse prevention.

Understanding how these platforms influence user behavior beyond simple access would provide a more nuanced view.

Response: Thank you for the suggestion, a section in the discussion line 654 that explores how the digital platforms promote education and counselling has been added, line 612-624.

11. Expand the discussion to consider how digital health platforms contribute to education, counseling, and responsible use of SRH products.

Response: Thank you. A section that discusses how Rocket Health digital platforms contribute to education and counselling has been added.

12. The study addresses a critical public health issue—SRH self-care among youth in Uganda—aligning with global efforts toward Universal Health Coverage.

Policymakers should ensure easy access for all in the digital age while also preventing misuse and the unregulated availability of medications with potential side effects without a physician's prescription.

Response: Thank you, this is noted.

Reviewer 2

2. **Overall Assessment:**
This manuscript explores the vital intersection of public health and technology, specifically the role of digital health platforms in enhancing access to sexual reproductive health (SRH) self-care among Ugandan youth. Given the significant challenges in accessing SRH services in sub-Saharan Africa, the study's context is highly relevant. However, the current draft requires substantial revisions to fully realize its potential. The analysis lacks depth, the discussion needs more engagement with existing literature, and the presentation of findings could be significantly improved.

Specific Recommendations:

Introduction:

Focus on Specific SRH Challenges: Clearly identify prevalent issues facing Ugandan youth, such as teenage pregnancy rates and unmet contraceptive needs. This will strengthen the rationale for the study.

Response: This has been rectified. The issues facing Uganda youth have been described on lines 63, 64, 65

Justify Digital Health's Role: Move beyond general statements about digital health potential. Analyze how these platforms can specifically address identified challenges, such as stigma and confidentiality concerns, and link mobile/internet penetration to the feasibility of interventions.

Response: This has been addressed in the introduction section, line 67-75

Contextualize Rocket Health: Provide background on Rocket Health's establishment, mission, and contributions to Uganda's digital health landscape to integrate it smoothly into the narrative.

Response: We have added a background on Rocket Health and its contributions to digital Health in Uganda, line 84-93

Methods:

Detail Mixed-Methods Integration: Clearly explain how qualitative and quantitative data were integrated and how they informed each other. Justify the choice of a cross-sectional design and discuss its limitations regarding causality.
Response: The intergration occurred at interpretation phase where the qualitative narratives where used to explain the quantitative trends forexample the easy and confidential ordering process of the E-commerce platform helped to explain the high high uptake of the contraception. The cross-sectional design was appropriate given the studies objective to explore digital health platforms in promoting access to SRH among youth in Uganda at a specific point in time and the novelty of the study area. We understand that the design limits the ability to infer causality and therefore future studies that could assess the sustained impact of digital health platforms on SRH outcomes.

Refine Sampling Strategy: Offer a detailed explanation of the purposive sampling strategy for qualitative data, including criteria for participant selection. For quantitative data, specify inclusion/exclusion criteria and the total number of records in the EMR system before filtering.

Response: This has been rectified in the sampling procedure section, line 161-191

Enhance Data Collection Details: Provide information on the development and content of interview guides, including whether they were pilot-tested. Describe the data extraction process from the EMR, specifying the variables collected.

Response: This has been fully described in the data collection section line 226-239

Specify Analytical Techniques: Clarify the qualitative analysis steps, including software used and how themes were identified. For quantitative analysis, detail the statistical tests applied.

Response: A clear description of the qualitative analysis steps has been done indicating how codes were categorized under the predefined themes. The process did not involve any software since the codes were assigned manually through an iterative process.

Results:

Improve Clarity and Presentation: Restructure the results section for better readability. Use tables and figures effectively to highlight key findings, ensuring all visuals have clear titles and labels.
Response: The has been rectified in the results section

Deepen Qualitative Presentation: Provide richer descriptions of qualitative findings supported by illustrative quotes. Connect these findings to quantitative results to show their complementary nature.

Response: This has been rectified with description and explanation

Clarify Costing Data: Offer a step-by-step explanation of how average expenditure was calculated, contextualizing these costs within the average income or cost of living in Uganda.

Response: This has been clarified, line 312-321

Discussion:

Engage with Existing Literature: Move beyond summarizing findings. Critically compare your results with other studies on digital health interventions in similar contexts and discuss the theoretical implications.
Response: This has been rectified with richer discussions, theoretical implications and comparisons from other studies. Forexample, line 462-495

Expand Limitations Discussion: Provide a comprehensive discussion of limitations, including the impact of the cross-sectional design on causal inference and potential biases in self-reported data.

Response: These have been discussed in the limitation section, line 627-647

Suggest Future Research Directions: Based on findings and limitations, recommend specific future research avenues, such as longitudinal studies to assess long-term impacts.

Response: Thank you for this comment. This was done under recommendations. We suggested a cost-effectiveness analysis to compare costs and benefits of utilizing digital platforms for sexual reproductive self-care, line 670-675

Overall Writing and Style:

Enhance Clarity and Conciseness: Improve writing clarity by minimizing jargon and using shorter sentences. Focus on logical organization to enhance the overall flow.

Response: This has been addressed

Standardize Referencing: Ensure consistent and accurate referencing throughout the manuscript, adhering to PLOS Digital Health's guidelines.

Response: We have standardized the referencing to the recommended referencing style (Vancouver)

Thank you for considering my feedback.
