# Supplementary material for: TELEHEALTH and digital health platforms in promoting access to sexual reproductive health self care among youth: A case of rocket health services in Uganda
Source: PLOS Digit Health. 2025 Oct 31;4(10):e0000770. doi: 10.1371/journal.pdig.0000770 (PMC12578243; doi:10.1371/journal.pdig.0000770)
Supplement: Attachment — Submitted filename: Response to Reviewers1.pdf [file pdig.0000770.s003.pdf]

Makerere University School of Public Health
Department Health Policy, Planning and Management

July 25 2025,

Haleh Ayatollahi

Section Editor

PLOS Digital Health,

Resubmission: ***TELEHEALTH and digital health platforms in promoting access to sexual reproductive health self care among youth: A case of Rocket health services in Uganda***

Dear Haleh Ayatollahi,

Thank you for the opportunity to revise our manuscript, "*TELEHEALTH and digital health platforms in promoting access to sexual reproductive health self-care among youth: A case of Rocket health services in Uganda*" PDIG-D-25-00072 for *PLOS* Digital Health.

We have addressed the reviewers' and editors' comments in our responses on the following pages and incorporated related changes into the manuscript. We appreciate the feedback and believe our manuscript is notably improved by our revisions following the reviewers' thoughtful evaluation.

This manuscript explores the different digital health platforms and how they promote access to reproductive health self-care among youth

These findings are pivotal for informing policymakers as they strategize policies, research, and resource allocation to Sexual Reproductive Health and self-care. We believe that publishing this work in PLOS Digital Health will significantly enhance its visibility and impact on policy decisions worldwide

We appreciate your consideration of this important manuscript and are eager for the opportunity to contribute to the body of research published by PLOS Digital Health.

Warm regards,

Vincent Ssenfuka
ssenfukavicent@gmail.com

Reviewer 1

1. The addition of Rocket Health's background improves clarity and aligns the platform with national digital health efforts

Response: Thank you

2. The expanded discussion on sildenafil use appropriately addresses concerns about misuse but would benefit from specifying if any provider safeguards are in place.

Response: This has been added in the discussion section, line 556 - 558. We have also discussed the different safeguards that could be applied to prevent misuse of such products.

3. The costing analysis is clearer and more policy-relevant; however, affordability in rural or lower-income youth is not sufficiently addressed.

Response: This was addressed in the discussion section where the cost findings were compared to Daily Wage of the lowest paid government worker (LPGW) in Uganda to ditermine affordabilty. The lowest paid government workers' wage was used to represent low income youth especially those in rural settings. Line 598 - 603.

4. Limitations are well detailed but should mention the exclusion of rural users, which affects the generalizability of findings.

Response: Thank you for the comment. We would like to clarify that rural users were not deliberately excluded as part of the study methodology. However, because the study relied on secondary data from Rocket Health's electronic medical records, the dataset did not capture rural users, resulting in their underrepresentation in our analysis. We have highlighted this limitation in the manuscript (line 682 - 683), noting that the absence of rural participants may affect the generalizability of the findings to more geographically diverse populations.
